# Light regulates chlorophyll biosynthesis via ELIP1 during the storage of Chinese cabbage

**DOI:** 10.1038/s41598-022-15451-9

**Published:** 2022-06-30

**Authors:** Tuoyi Wang, Sijia Liu, Shaonan Tian, Tianyi Ma, Wei Wang

**Affiliations:** 1 College of Food and Biological Engineering, Qiqihar University, Qiqihar, 161006 China; 2 College of Life Sciences, Agriculture and Forestry, Qiqihar University, Qiqihar, 161006 China

**Keywords:** Plant sciences, Light responses, Plant molecular biology, Plant stress responses

## Abstract

Chlorophyll loss is a major problem during green vegetable storage. However, the molecular mechanism is still unclear. In this study, a 21 days of storage experiments showed chlorophyll content was higher in light-stored Chinese cabbage (*Brassica chinensis* L*.*) leaves than those in dark-stored samples. Transcriptome analyses were performed on these samples to determine the effects of light. Among 311 differentially expressed genes (DEGs), early light-induced protein 1 (ELIP1) was identified as the main control gene for chlorophyll synthesis. Tissues and subcellular localization indicated that ELIP1 was localized in the nucleus. Motifs structure analyses, chromatin immunoprecipitation (ChIP) assays, luciferase reporter assays, and overexpression experiments demonstrated that ELIP1 regulated the expressions of genomes uncoupled 4 (*GUN4*), Glutamyl-tRNA reductase family protein (*HEMA1*), and Mg-protoporphyrin IX methyltransferase (CHLM) by binding to G-box-like motifs and affected chlorophyll biosynthesis during the storage of Chinese cabbage. It is a possible common tetrapyrrole biosynthetic pathway for chlorophylls, hemes, and bilin pigments in photosynthetic organisms. Our research also revealed that white light can be used as a regulatory factor to improve the storage ability and extent shelf life of Chinese cabbage.

## Introduction

Chinese cabbage (*Brassica chinensis* L*.*) belongs to cruciferous plants, which is one of the most important autumn vegetable varieties^1–3^. Chlorophyll content sometimes represents the storage quality of green vegetables^4,5^. Dark-adapted leaf senescence caused by chlorophyll loss is an essential problem during vegetable storage^6^. Some studies have revealed that light storage can prevent the leaf senescence and maintain the total chlorophyll content higher than dark storage^7^. However, the molecular mechanism is still unclear.

In green plants, chlorophyll biosynthesis starts with glutamyl-tRNA and ends with chlorophyll b synthesis^8^, which is controlled by multiple genes. For example, genomes uncoupled 4 (*GUN4*) is involved in the control of tetrapyrrole synthesis during the chlorophyll biosynthesis as a regulatory sub-unit of Mg-chelatase^9^, glutamyl-tRNA reductase family protein (*HEMA1*) is acutely dependent on phytochrome signaling and permitted the tetrapyrrole synthesis for the accumulation of chlorophyll^10^, and Mg-protoporphyrin IX methyltransferase (CHLM) plays a crucial role in chlorophyll biosynthesis and converts Mg-protoporphyrin IX to Mg-protoporphyrin IX monomethyl ester^11–14^. But recent research indicates that the chlorophyll a/b-binding (CAB) protein family is a crucial precursor signal during chlorophyll biosynthesis and metabolism^15,16^. At least 30 CAB family proteins have been found in *Arabidopsis thaliana*^17,18^, including photo-system protein^19^, one-helix protein^20^, and early light-induced proteins (ELIPs)^21^.

As a photoinduced protein, ELIPs broadly exists in various plants^22^. Chinese cabbage has two ELIPs alleles*,* namely ELIP1 and ELIP2^23^. ELIP1 is nucleus-encoded protein that is synthesized on cytoplasmic ribosomes, then transfers to the chloroplast, and anchors to thylakoid membranes through three transmembrane domains^22–25^. ELIP1 can be expressed transiently in response to light in etiolated plants^26^. Under moderate light intensities conditions, ELIP1 only plays a slight role in the chlorophyll accumulation^27^. However, ELIP1 expression and chlorophyll accumulation dramatically decrease at low temperature and low light intensity^28^. In brief, the physiological function of ELIP1 may be related to the regulation of chlorophyll biosynthesis, but molecular mechanism on regulatory pathway has barely been reported, especially during the storage of green vegetable. In this study, we demonstrated some molecular mechanism on the light regulation of chlorophyll biosynthesis via ELIP1 during the storage of Chinese cabbage.

## Results

### Screening for chlorophyll regulatory genes during the storage of Chinese cabbage

**Figure 1:**
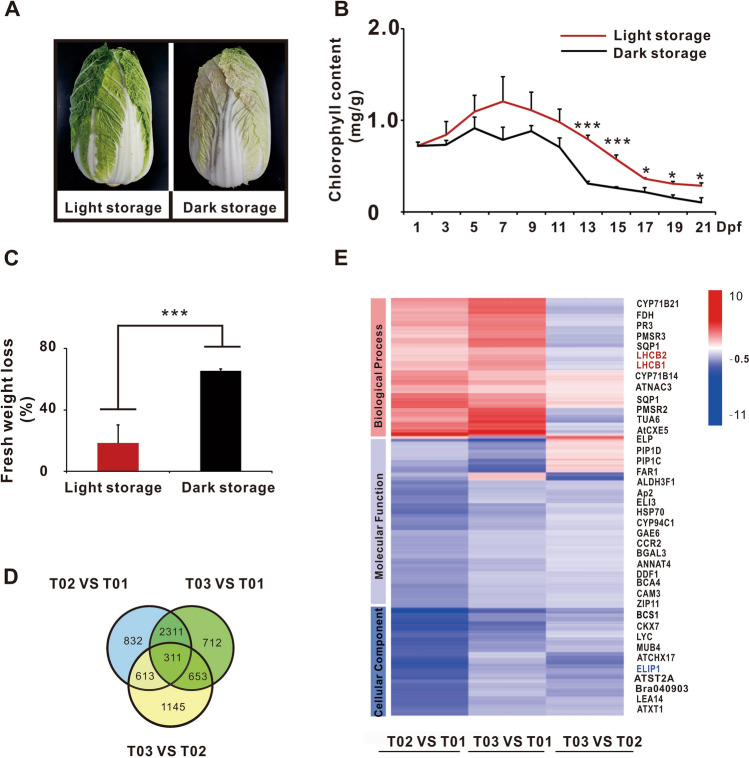
Screening for chlorophyll regulatory genes during the storage of Chinese cabbage. (**A**) Changes in appearance of Chinese cabbage. After 21 days, Chinese cabbage leaves under light storage condition were still green, but the leaves under dark storage condition lost most of their green pigment. (**B**) Chlorophyll content. Chlorophyll content was higher in light-stored samples than in dark-stored samples. (**C**) Percentage of fresh weight loss. Light-stored Chinese cabbage has a lower percentage fresh weight loss than the dark-stored ones after 21 days storage. (**D**) Venn diagram of DEGs. TS01 was fresh Chinese cabbage samples as control, TS02 was the 10th day Chinese cabbage samples under dark storage, and TS03 was the 20th day Chinese cabbage samples under dark storage. (**E**) Heat map showing up-regulated and down-regulated genes during the storage of Chinese cabbage. ELIP1, LHCB1, and LHCB2 are labeled on the right side of the heat map. Red color represents the up-regulated genes, and blue color represents the down-regulated genes. Asterisks indicate a significant difference compared to control negative as analyzed by Dunnett, one-way ANOVA. (*p < 0.05; **p < 0.01; ***p < 0.001). All error bars are expressed as SEM.

After 21 days of storage, Chinese cabbage leaves stored under white light condition were still green, whereas those under dark condition lost most of their green pigment and become senescence (Fig. 1A). Chlorophyll content was higher in light-stored samples than those in dark-stored samples as revealed in Fig. 1B. The percentage of fresh weight loss of light-stored cabbage samples was also much lower than those of dark-stored samples (Fig. 1C). These results showed that light was very important to maintain the chlorophyll content and fresh weight during the storage of Chinese cabbage.

Transcriptome analyses were performed on Chinese cabbage samples to explore the molecular mechanisms. TS01 was fresh Chinese cabbage samples as control, TS02 samples were obtained on the 10th day of dark storage, and TS03 samples were obtained on the 20th day of dark storage. The results uncovered 3,687 DEGs at T02 versus T01 (Fig. 1D, Supplementary Fig. S1), 3,621 DEGs at T03 versus T02 (Fig. 1D, Supplementary Fig. S2), 2,472 DEGs at T02 versus T01 (Fig. 1D, Supplementary Fig. S3), and 311 common DEGs at T03 versus T02 versus T01 (Fig. 1D,E). Gene Ontology (GO) and Kyoto Encyclopedia of Genes and Genomes (KEGG) were applied for the analyses of these DEGs and revealed the genes involved in biological process, molecular function, and cellular component that were markedly altered in the storage of Chinese cabbage (Supplementary Figs. S1–S3). Clustering analysis suggested that more genes were down-regulated in dark-stored Chinese cabbage (Fig. 1E); therefore, light is a possible regulatory factor in the expression of these down-regulated genes. Among 311 DEGs, three CAB family members, namely, *ELIP1*, *LHCAB1.1*, and *LHCAB2.1* may be closely related to the changes in chlorophyll content during the storage of Chinese cabbage (Fig. 1E, Supplementary Table S3).

### Light regulation experiment

**Figure 2:**
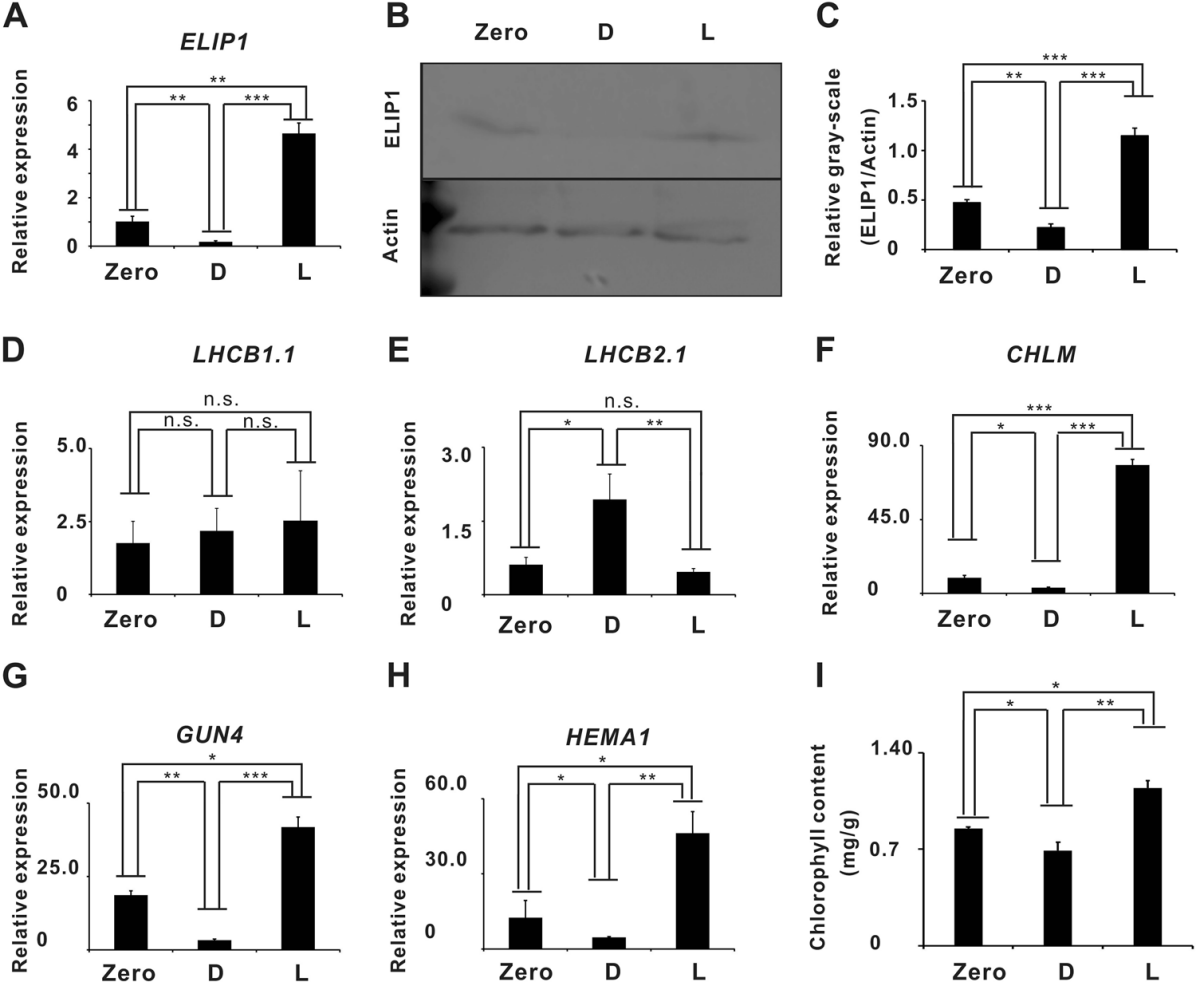
Light regulation experiment. (**A**) *ELIP1* expression. (**B**) Western-blot shows that ELIP1 expression was higher in the samples exposed to light but hardly detected the samples stored in the dark. (**C**) Quantitative analysis of Western blot strips by Image J software. Light can promote the expression of ELIP. (**D**) *LHCB1.1* expression. (**E**) *LHCB2.1* expression. (**F**) *CHLM* expression. (**G**) *GUN4* expression. (**H**) *HEMA1* expressions. (**I**) Chlorophyll content. Fresh Chinese cabbage samples were used as the control and marked as Zero, the samples stored under light condition were marked as L, and the samples stored under dark condition were marked as D. Asterisks indicate a significant difference compared to control negative as analyzed by Dunnett, one-way ANOVA. (*p < 0.05; **p < 0.01; ***p < 0.001). All error bars are expressed as SEM.

Transcriptome analysis revealed that light, as an important factor, had potential regulatory effects on chlorophyll content and gene expression. Further qRT-PCR results showed that *ELIP1* was down-regulated in the dark but up-regulated under light exposure (Fig. 2A). The results of western blot indicated that ELIP1 signal was increased after light exposure, but was hardly detected after dark storage (Fig. 2B,C). Expression levels of *LHCBA1.1* and *LHCBA2.1* in light exposure had no substantial changes (Fig. 2D,E), but *LHCBA2.1* was up-regulated in dark condition (Fig. 2D). We analyzed the expression of ELONGATED HYPOCOTYL 5 (HY5) and PHYTOCHROME INTERACTING FACTOR 3 (PIF3) and the results displayed elevated HY5 and decreased PIF3 in the dark (Supplementary Fig. S4A,B). This was consistent with the results of Cazzonelli et al.^28^. PIF3 and HY5 may be as a retrograde signal to regulating LHCBA2.1 expression to maintain the stability of chlorophyll content during dark storage in Chinese cabbage. Chlorophyll synthesis genes *GUN4, HEMA1* and *CHLM* were prominently increased under light condition (Fig. 2F–H), but dramatically decrease under dark condition (Fig. 2F–H). In addition, we also found that the chlorophyll content increased significantly under light condition, but decreased slightly under dark condition after a 24-h light regulation experiment (Fig. 2I). These results were consistent with the expression trends of *ELIP1* (Fig. 2A) and suggested that *ELIP1* could be the control gene for chlorophyll biosynthesis during the storage of Chinese cabbage.

### Tissue and subcellular localization analyses of ELIP1

We performed the tissue and subcellular localization with immunohistochemistry (IHC) and ELIP1-CFP fusion protein to investigate the localization of the ELIP1 protein in Chinese cabbage. The completely overlapped fluorescence signals of DAPI and IHC in tissue sections demonstrate that ELIP1 was localized near the nucleus (Fig. 2A–C). ELIP1-CFP fusion protein was also localized in the nucleus by comparing the overlapping areas of DAPI signal in the protoplast cells (Fig. 2B–D). In the 40 × field, ELIP1-CFP signal was showed in nucleus (white arrow) and cell wall of Chinese cabbage protoplasts (Fig. 2E–G). These findings indicated that ELIP1 was localized in the nucleus, which provided a molecular basis for its regulation on chlorophyll synthesis genes.

### ELIP1 regulates *GUN4*, *HEMA1*, and *CHLM* via the G-box-like motifs

As a *cis*-acting DNA regulatory element, the G-box (CACGTG) can be bound to proteins known as G-box factors (GBFs) in a context-specific manner in plant genomes^30^. The motif structure analyses indicated that there were two typical G-box motifs in the promoter regions of *ELIP1*, nine G-box-like (CACGTN) motifs in the promoter regions of *CHLM*, one G-box-like motif in the promoter regions of *GUN4*, and one G-box-like motif in the promoter regions of *HEMA1* (Fig. 4A)*.* The binding relationship between ELIP1 and *CHLM*, *GUN4*, and *HEMA1* was investigated by ChIP assays. The results revealed that ELIP1 could bind to G-box-like motifs in *CHLM* (− 1034, Fig. 4B,C), *CHLM* (− 1089, Fig. 4B,D), *GUN4* (− 1586, Fig. 4B,E), and *HEMA1* (− 168, Fig. 4B,F).

Subsequently, *ELIP1* cDNA was cloned into the pCambia1301 vector. Promoter sequence of *CHLM*, *GUN4*, and *HEMA1*, including ELIP1 binding sites and 5′-untranslated region, was cloned into pGreenII 0800-Luc vectors and transformed into agrobacterium. The luciferase gene reporter assays were performed three days after agro-inoculation. The results demonstrated that luciferase activities mediated by G-box-like motifs on *CHLM-Luc, GUN4-Luc*, and *HEMA1-Luc* were activated by pCambia1301-ELIP1 (Fig. 4G–I). A construct was designed wherein the G-box-like motifs were deleted to further investigate their potential role. CHLM-mut-Luc, GUN4-mut-Luc, and HEMA1-mut-Luc lost luciferase activities when added to the luciferase system with pCambia1301-ELIP1 (Fig. 4G–I). In summary, G-box-like motifs played key roles on the transcription regulation of ELIP1 on *CHLM, GUN4*, and *HEMA1*.

### *ELIP1* overexpression increased the expression levels of *CHLM, GUN4*, and *HEMA1* and the biosynthesis of chlorophyll

Positive *A. tumefaciens* EHA105 colonies containing pBI121–ELIP1 vectors were employed to infect Chinese cabbage leaves for *ELIP1* overexpression to verify the chlorophyll changes caused by the downregulation of ELIP1 in dark-stored Chinese cabbage (Fig. 1A). The ELIP1 overexpression transgenic line (EO line) showed a higher level of *ELIP1* expression, whereas the WT and control exhibited lower *ELIP1* expression levels (Fig. 5A). Similarly, *CHLM* (Fig. 5B), *GUN4* (Fig. 5C), and *HEMA1* (Fig. 5D) all showed higher expression levels in the EO line. We also measured the chlorophyll content in the WT, control, and EO line. The results showed that the chlorophyll content was higher in the EO line than that in the control group (Fig. 5E). We also found that there was no significant difference on chlorophyll content between the WT and EO line, which may be due to the inevitable chlorophyll loss in the working solution during leaf infection in the EO line (Fig. 5E). These results demonstrated that the *ELIP1* gene was successfully introduced into the Chinese cabbage plants and substantially increased the expression levels of *CHLM*, *GUN4*, and *HEMA1*, as well as chlorophyll biosynthesis.

## Discussion

In this study, a 21 days of storage experiments showed Chinese cabbage leaves stored under white light were still green, whereas those under dark storage lost most of their green pigment and became senescence. The chlorophyll content was higher in light-stored Chinese cabbage leaves than those in dark-stored samples. Transcriptome analyses showed that ELIP1 may be the main control gene for chlorophyll synthesis during the storage of Chinese cabbage.

Light can improve vegetable quality at harvest and postharvest, but the molecular mechanism has not been revealed. In this study, we discussed some molecular mechanism on the light regulation of chlorophyll biosynthesis via ELIP1 during the storage of Chinese cabbage. ELIP1was closely related to chlorophyll biosynthesis and seed germination^27,29^. The results showed that chlorophyll content and *ELIP1* expression in light-stored cabbage samples were much higher than those of dark-stored samples (Fig. 1A,B). *CHLM*, *GUN4*, and *HEMA1* were upregulated under light exposure (Fig. 2F–H) and in the EO line (Fig. 5B–D). Combined with previous findings, our study revealed synergistic regulations between *ELIP1*, chlorophyll content, *CHLM*, *GUN4*, and *HEMA1* during the storage of Chinese cabbage.

**Figure 3:**
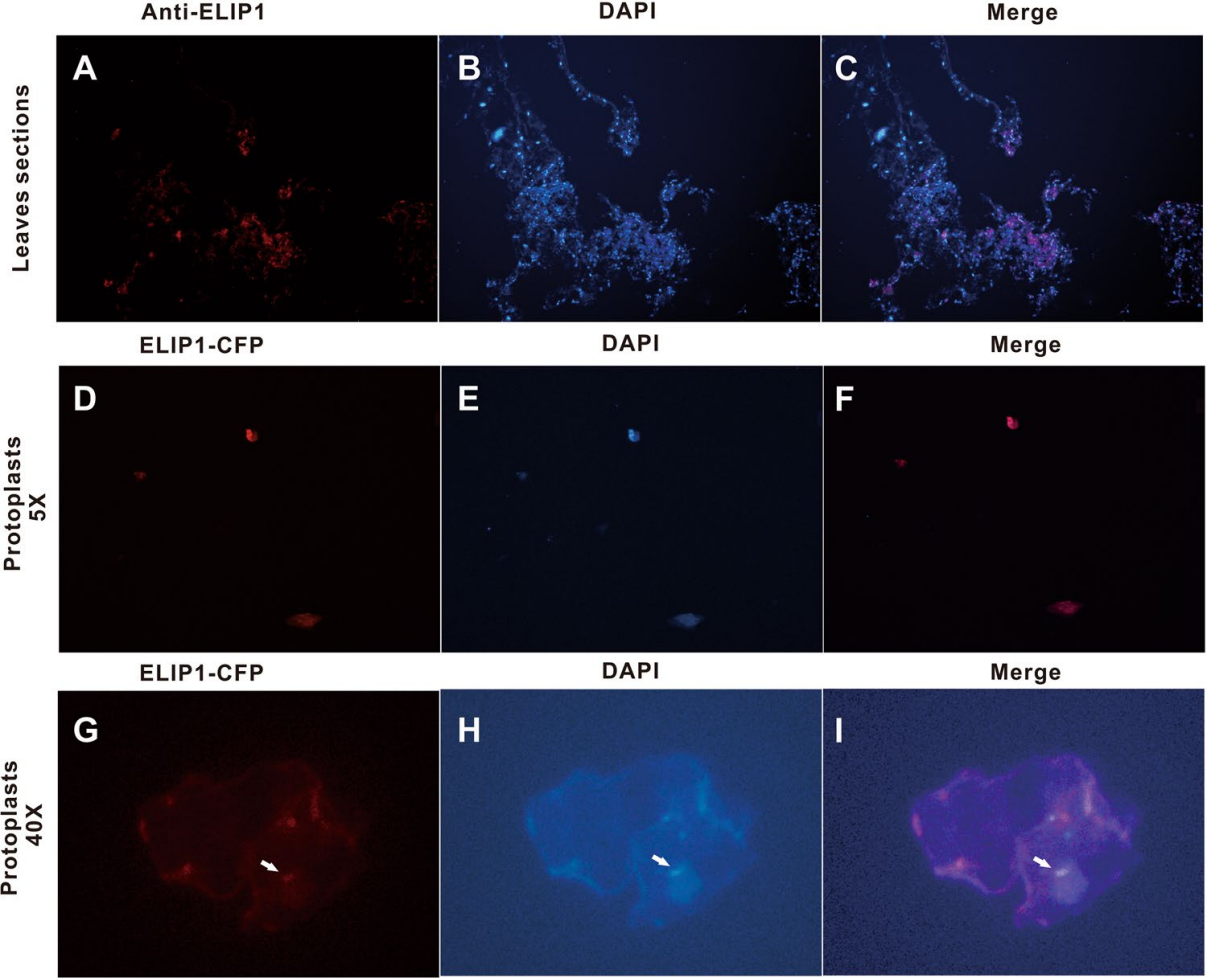
Tissue and subcellular localization of ELIP1. (**A**) Tissue localization of ELIP1 on the upper epidermis of Chinese cabbage leaf. (**B**) DAPI staining. (**C**) Merged. (**D**) Transfection of ELIP1-CFP fusion protein into Chinese cabbage protoplasts. The microscope eyepieces was in quintuple vision. (**E**) DAPI staining in Chinese cabbage protoplasts. The microscope eyepieces was in quintuple vision. (**F**) Merged. The microscope eyepieces was in quintuple vision. (**G**) ELIP1-CFP fusion protein in nucleus of Chinese cabbage protoplasts. ELIP1-CFP was marked by the white arrow. The microscope eyepieces was in 40 times vision. (**H**) DAPI staining in nucleus of Chinese cabbage protoplasts. ELIP1 in nucleus was pointed by the white arrow. The microscope eyepieces was in 40 times vision. (**I**) Merged. ELIP1 in the nucleus was pointed by the white arrow.

**Figure 4:**
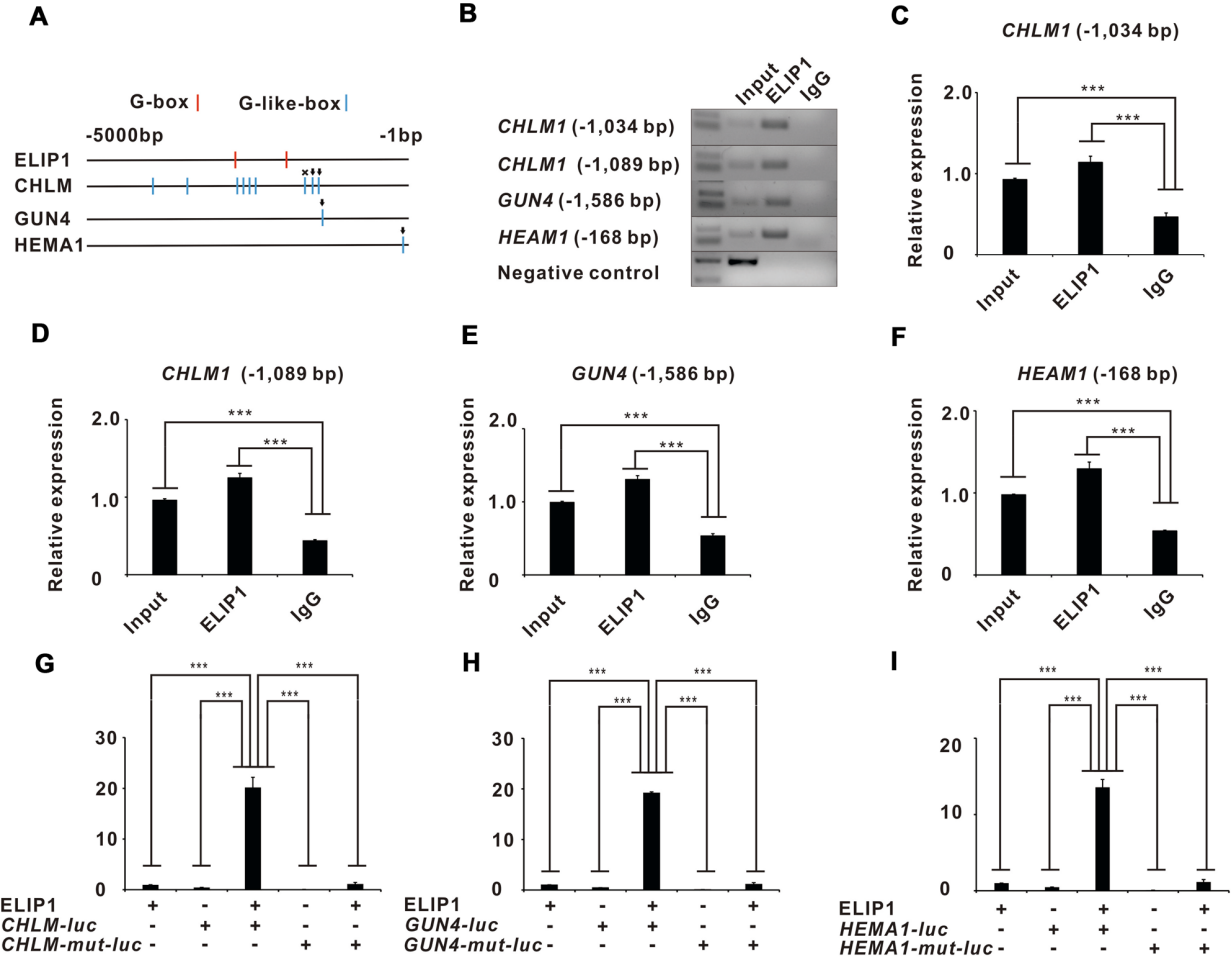
ELIP1 regulates *CHLM*, *GUN4*, and *HEMA1* via the G-box-like motifs. (**A**) Sketch map of G-box (in red) and G-box-like motifs (in blue) in gens promoter. The ELIP1-binding G-box-like motifs were marked with black arrows, and the non-ELIP1-binding G-box-like motifs were marked with the “×” symbol. (**B**) Gel electrophoresis of ChIP assays. (**C**) Higher binding strength of ELIP1 at the site of *CHLM* (− 1034) than Input and IgG. The binding strength was the ratio of ChIP signal to the normalized Input signal. (**D**) Higher binding strength of ELIP1 at the site of *CHLM* (− 1089) than Input and IgG. The binding strength was the ratio of ChIP signal to the normalized Input signal. (**E**) Higher binding strength of ELIP1 at the site of *GUN4* (− 1586) than Input and IgG. The binding strength was the ratio of ChIP signal to the normalized Input signal. (**F**) Higher binding strength of ELIP1 at the site of *HEMA1* (− 168) than Input and IgG. The binding strength was the ratio of ChIP signal to the normalized Input signal. (**G**) Luciferase reporter assays showed that C*HLM* was directly regulated by ELIP1 through G-box-like motifs, but luciferase activities were inhibited when the binding sites were mutated. (**H**) Luciferase reporter assays showed that *HEMA1* was directly regulated by ELIP1 through G-box-like motifs, but luciferase activities were inhibited when the binding site was mutated. (**I**) Luciferase reporter assays showed that *GUN4* was directly regulated by ELIP1 through G-box-like motifs, but luciferase activities were inhibited when the binding site was mutated. Asterisks indicate a significant difference compared to control negative as analyzed by Dunnett, one-way ANOVA. (*p < 0.05; **p < 0.01; ***p < 0.001). All error bars are expressed as SEM.

Like other eukaryotes, the expression of plant genes is regulated at the chromosome, transcription, post-transcription, translation, and post-translation levels, among which transcriptional regulation is of great importance^8^. The transcription regulation of plant genes plays its role through the interactions between regulatory factors and the specific sequences of promoter regions (*cis*-acting elements). Plants have various types of *cis*-acting elements, such as G-box^31,32^, E-box^33^, D-box^34^, and F-box^35^. Among them, G-box (CACGTG), a typical motif with six base palindrome sequences, is broadly distributed in plants and plays an essential role on the photoinduced regulation of plant genes^36^. Figure 4A revealed the presence of a large number of G-box and G-box-like motifs (CACGTN) in the promoter regions of *ELIP1*, *CHLM*, *HEMA1*, and *GUN4*. Transcriptional regulation by promoter-targeted protein needs to be undertaken in the nucleus to promote transcription activation or silencing^37^. Previous studies showed that mature ELIPs in pea was transferred to the chloroplast and anchored to thylakoid membranes through three transmembrane domains^24^. However, these studies did not suggest whether ELIP1 exists in the nucleus. Therefore, proving that ELIP1 is located in the nucleus of Chinese cabbage plants is important. In our study, the tissue and subcellular localization clearly showed that ELIP1 located in the nucleus of plant tissues and protoplasts in Chinese cabbage (Fig. 3). These findings provide a very strong evidence that ELIP1 could regulate downstream genes through interactions with *cis*-acting elements.

**Figure 5:**
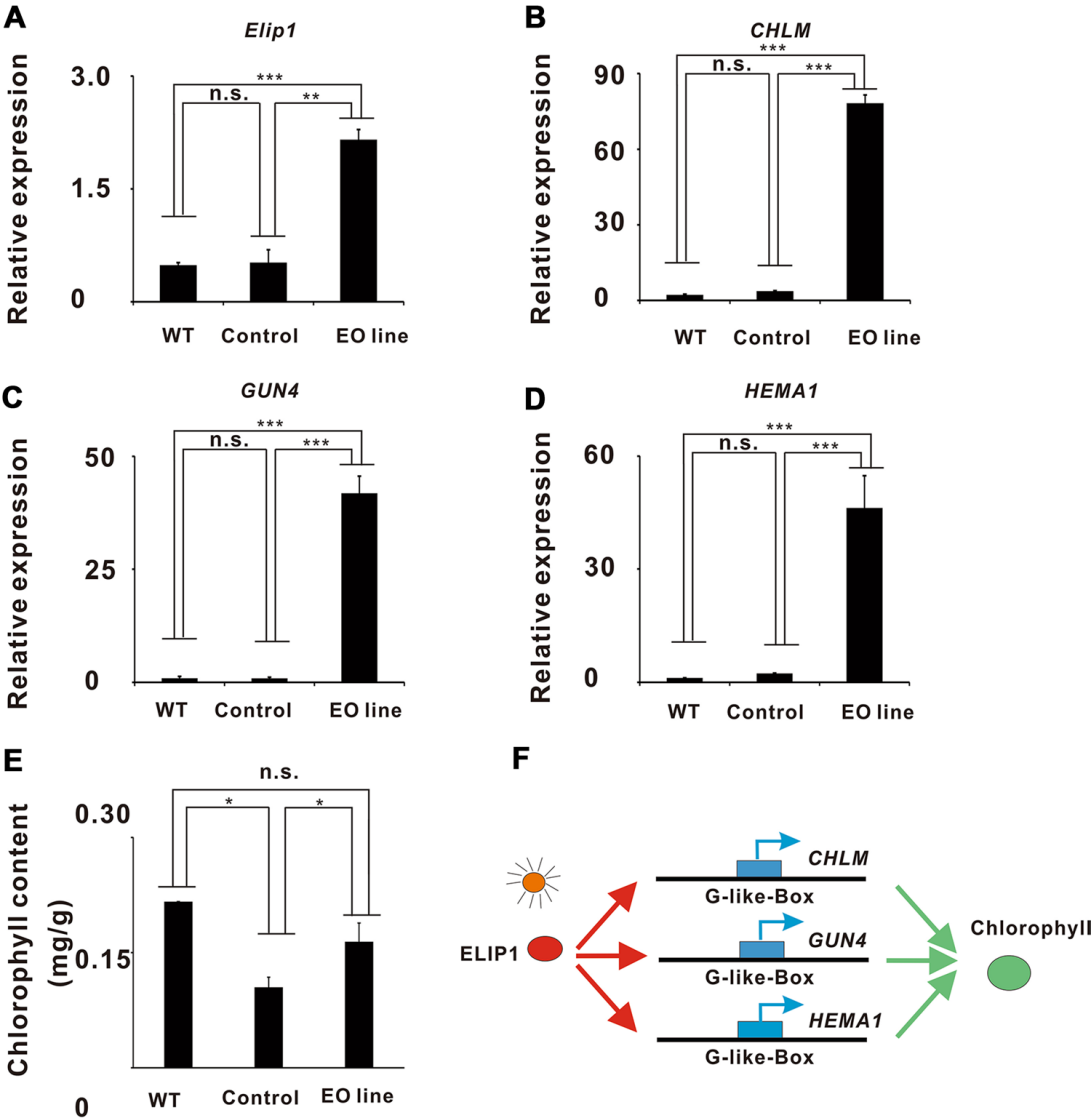
*ELIP1* overexpression increased the expression levels of *CHLM*, *GUN4*, and *HEMA1* and chlorophyll content. (**A**) The expression of ELIP increased after the transfection of pBI121-ELIP1 vectors into Chinese cabbage leaves. (**B**) The expression of *CHLM* increased in the EO line. (**C**) The expression of *GUN4* increased in the EO line. (**D**) The expressions of *HEMA1* increased in the EO line. (**E**) Chlorophyll content of the EO line was higher than that of the control group, but the mean value was slightly lower than that of the WT group. (**F**) Model designed for chlorophyll regulation in postharvest Chinese cabbage. The ELIP1 protein regulated *CHLM*, *GUN4,* and *HEMA1* via G-box-like motifs and affected chlorophyll biosynthesis during Chinese cabbage storage. EO line is the ELIP1 overexpression transgenic line, negative *A. tumefaciens* EHA105 colonies were used as the control, and “Beijing new No. 3” Chinese cabbage leaves were used as wild type (WT). Asterisks indicate a significant difference compared to control negative as analyzed by Dunnett, one-way ANOVA. (*p < 0.05; **p < 0.01; ***p < 0.001). All error bars are expressed as SEM.

ChIP assays and luciferase experiments showed that ELIP1 regulated the luciferase activities of *CHLM*, *GUN4*, and *HEMA1* by directly binding with G-box-like motifs (Fig. 4B–F). Figures 1, 2, and 5 showed that *ELIP1*, *CHLM*, *GUN4*, and *HEMA1* were directly correlated to the chlorophyll content during the storage of Chinese cabbage. However, chlorophyll biosynthesis in higher plants contains a series of enzymatic reactions and a great number of genes. In fact, the motif structure analyses indicated that there was a typical G-box motif in the promoter regions of PROTOCHLOROPHYLLIDE OXIDOREDUCTASE C (POR C)^28^. We checked the expression levels of POR C under dark/light storage and found a decreased expression in dark and increased expression in light (Supplementary Fig. S4C). However, the results of luciferase and ChIP assay showed that ELIP1 could not drive the expression of POR C-luc and bind to the promoter region of POR C (Supplementary Fig. S4D–E), so we speculated that POR C was regulated by light but not by ELIP1. In addition, there is a POR gene (Bra010646) in Chinese cabbage, but the functional annotation is not clear, so we did not detect the expression of POR. Whether other genes were regulated by ELIP1 still needs further investigation. Previous studies have shown that photosynthetic organisms synthesize chlorophyll, heme, and bilin pigments via a common tetrapyrrole biosynthetic pathway^11^. Our study also provided a possible molecular mechanism for this common pathway (Fig. 5F).

Studies have shown that photosynthetic carbon assimilation can increase CO_2_ fixation and biomass yield in tobacco^38^. Moreover, due to the lack of photosynthesis during dark storage, Chinese cabbage leaves often rot and lost weight, which was also calculated in the fresh weight loss. Therefore, the percentage of fresh weight loss in light-stored cabbage samples was much lower than those of dark-stored samples (Fig. 1C). The results suggested that light can maintain the green and weight and had good freshness-keeping effects on Chinese cabbage (Fig. 1A–C). In short, this study suggests that light can regulate chlorophyll biosynthesis via ELIP1 and prolong the shelf life of Chinese cabbage.

In conclusion, our study provided some molecular mechanism about vegetable chlorophyll biosynthesis during storage. ELIP1 regulates *CHLM*, *HEMA1*, and *GUN4* via the G-box-like motifs to affect chlorophyll biosynthesis during the storage of Chinese cabbage.

## Materials and methods

### Plant materials, storage conditions, and leaves sampling

Chinese cabbage (BeijingNew No. 3) samples came from Merris vegetable base in Qiqihar, Heilongjiang Province, China. “Beijing New No. 3” cabbage is the F1 of self-incompatibility lines 832,172 cabbage and 84,427 cabbage. “Beijing New No. 3” cabbage was selected and bred by the Vegetable Research Center of Beijing Academy of Agriculture and Forestry, Beijing, China. We have permission to collect the plant samples used in the study.

These samples for storage were kept in a storage cabinet with a temperature of 2–6 °C and a humidity of 60–70% RH. In addition to light intensity, dark storage condition was the same as light storage. The light intensity of light storage was about 1100–1700 lx, but the light intensity of dark storage was reduced to about 0–3 lx.

Multi-point sampling collection was selected for the leaves sampling. Each leaf had 1–2 sampling points, and a maximum of 2 g leaves were collected each point. The leaves were washed three times with double distilled water and dried with absorbent paper for further experiments. Leaves sampling needs to be repeated 3–6 times for each experiment.

### Chlorophyll content and the percentage fresh weight loss

Chlorophyll content was measured with the acetone methods. First, about 2 g Chinese cabbage leaves were cut into small pieces, placed into a 50-ml centrifuge tube, added with 5 ml of 80% acetone, and ground with a grinding pestle. The supernatant was retained for further experimental use. Second, the supernatant was centrifuged at 10,000 r/min for 1 min. The supernatant (2 ml) was added with 18 ml of ethanol to prepare a 20 ml working fluid. Finally, the working fluid was analyzed at 645 and 663 nm with a 721 spectrophotometer. Chlorophyll content was calculated as the sum of chlorophyll a and chlorophyll b.

Twelve fresh Chinese Cabbage were selected, removed sediment and impurities, wiped clean, wrapped around fresh-keeping film, weighed (Initial mass), and put into the storage cabinet mentioned above. There were six cabbages in the light layer and six cabbage in the dark layer. Twenty-one days later, these cabbages were taken out, the rotten tissues were removed, and the final weigh was gotten (final mass). The percentage of fresh weight loss = ((initial mass − final mass)/initial mass)) × 100.

### RNA quantification for transcriptome analysis

Total RNA was extracted from the Chinese cabbage leaves on the first day (TS01, fresh samples), the 10th day (TS02), and the 20th day (TS03) using Trizol (Invitrogen) for transcriptome sequencing according to the manufacturer's protocol. Each sample needed about 100 mg leaves. The sample was cut into small pieces, placed into a 2-ml centrifuge tube, added with 500 µl of Trizol, and ground with a grinding pestle. First-strand cDNAs were synthesized using random hexamer primers and M-MuLV reverse transcriptase (RNase H-), and second-strand cDNA was synthesized using DNA polymerase I and RNase H. Remaining overhangs was converted into blunt ends via exonuclease/polymerase activities. The 3′ ends of DNA fragments were adenylated, and NEBNext Adaptor with a hairpin loop structure was ligated to prepare for hybridization. The library fragments were purified with the AMPure XP system (Beckman Coulter) to select cDNA fragments with preferentially 240 bps in length. Then, 3 μl of USER enzyme was used with size-selected, adaptor-ligated cDNA at 37 °C for 15 min followed by 5 min at 95 °C before polymerase chain reaction (PCR). PCR was performed using a high-fidelity DNA polymerase, Universal PCR primers, and Index (X) primer. The PCR products were purified, and library quality was assessed on the Agilent Bioanalyzer 2100 system.

### Deep sequencing-based transcriptome analysis

Clean reads were mapped to the Chinese cabbage reference genome (ftp://brassicadb.org/bra'chrome'v1.5/) by TopHat v2.0.13. Gene expression was calculated by Cufflinks. Gene expression levels were normalized by reads per kilobase of transcript per million mapped reads. Differentially expressed genes (DEGs) were identified by the DESeq R package (1.10.1), and the resulting *p* values were adjusted using the Benjamini and Hochberg’s approach for controlling the false discovery rate. Genes with adjusting *p* < 0.05 found by DESeq were assigned as differentially expressed.

### Total RNA extraction for quantitative real-time PCR (qRT-PCR)

Total RNAs were extracted from 50 mg Chinese cabbage using TRIzol^®^ reagent (Ambion). The sample was cut into small pieces, placed into a 1.5-ml centrifuge tube, added with 500 µl of Trizol, and ground with a grinding pestle. The purified total RNAs (2 µl) were reverse transcribed into cDNAs. qRT-PCR was performed with a Bio-Rad Laboratories instrument with the TB-Green detection system. Primers (Comate Bioscience Technology) used for qRT-PCR are listed in Supplementary Table S1. All results were normalized to the expression level of the housekeeping gene *ACTIN2*. qRT-PCR results are shown as a relative expression level calculated using the 2^−ΔΔ^ CT method. *p* values were calculated with one-way ANOVA test.

### Light regulation experiment

Fresh Chinese cabbage samples were separately stored in white light (1100–1700 lx) and dark (0–3 lx) for 24 h in the 4 °C storage cabinet. Then, about 2 g leaves were immediately harvested with light avoidance for qRT-PCR and chlorophyll content analysis. qRT-PCR was performed to analyze the gene expression under light and dark conditions. Fresh Chinese cabbage samples were used as the control and marked as Zero, the light-stored samples were marked as L, and the dark-stored samples were marked as D (Fig. 2).

### Western blot

About 20 mg samples were collected, chopped, and ground with 200 μl of lysis buffer (Beyotime Biotechnology). The precipitates were mixed with sodium dodecyl sulfate (SDS)–polyacrylamide (PA) gel electrophoresis sample buffer in a ratio of 4:1, boiled for 3–5 min, and run on 12% SDS-PA gel. The western blot was conducted with anti-ELIP1 (1:3000) and anti-ACTIN-plant (1:800) rabbit polyclonal antibodies. The quantitative analysis of the Western blot strips was carried out by Image J (National Institutes of Health).

### Tissue localization of ELIP1 with immunohistochemistry (IHC)

Chinese cabbage samples were fixed in 4% paraformaldehyde at 4 °C for 1–2 days, dehydrated with different concentrations of ethanol gradient elution, and made transparent with xylene solution. The samples were embedded with optimum cutting temperature compound and cut into 6–8 μm sections. The sections were baked under 80 °C for 30 min, then washed with PBS solution, permeated with 0.1% Triton X-100 for 10 min, and blocked with 3% BSA for 30 min. The sections were incubated with the primary antibody of polyclonal rabbit anti-ELIP1 under 4 °C for one night, then washed with PBS solution, and incubated with the secondary antibody, fluorescein isothiocyanate-conjugated donkey anti-rabbit IgG, under 37 °C in the dark for 1 h. Finally, the sections were softly rinsed with PBS and stained with 4,6-diamidino-2-phenylindole (DAPI) for about 10 min. The stained upper epidermis of cabbage leaf were observed under a fluorescence microscope (Olympus, BX53).

### Cloning and subcellular localization of ELIP1

ELIP1 cDNA (603 bp) was amplified by PCR and cloned into pBI121-NLS-CFP vector (MiaoLing Plasmid Sharing Platform) with XbaI and BamHI sites. The recombinant pBI121–NLS–CFP plasmids were introduced into the protoplast cells from the leaves of Chinese cabbage using PEG400 solution. The protoplast cells were cultivated on MS plates overnight at 25 °C (about 16 h) and observed under a fluorescence inverted microscope (Olympus, IX53).

### Generation of ELIP1 overexpression line in Chinese cabbage

ELIP1 cDNA (603 bp) was joined to pBI121 vector with XbaI and BamHI sites. The recombinant vector pBI121-ELIP1 was transformed into *Agrobacterium tumefaciens* EHA105 using the freeze–thaw method. Positive *A. tumefaciens* EHA105 colonies containing PBI121-ELIP1 were employed for the transformation of cabbage leaves using the floral dip method (n = 110). Negative *A. tumefaciens* EHA105 colonies were used as the control (n = 110). “Beijing new No. 3” Chinese cabbage leaves were used as wild type (n = 110). In particular, these cabbage leaves were cut into circles by the puncher, and their diameter was about 6–8 mm.

qRT-PCR was performed with the RNA samples from the wild type (WT), control and ELIP1 overexpression transgenic line (EO line) cultured for 24 h with MS medium to assay the transient expression of *ELIP1*, *CHLM*, *GUN4*, and *HEMA1*. The chlorophyll content was measured using the acetone method as described above.

### Chromatin immunoprecipitation (ChIP)

ChIP assays was performed according to the manufacturer’s protocol (56383 SimpleChIP^®^ Plus Sonication ChIP Kit 4 and RT Reagents, Cell Signaling Technology). Briefly, 100–150 mg Chinese cabbage leaves were cut into small pieces and divided and cross-linked with 1% formaldehyde at room temperature for 30 min. The crosslinking was terminated by adding 100 μl of 10 × glycine, and then the sample was mixed and incubated on ice for 5 min, washed with the mixture of PBS and 200 × protease inhibitor cocktail (PIC, twice every 10 min), and centrifuged. The supernatant was removed, and the tissue was resuspended in 1 ml mixture of 1 × ChIP sonication cell lysis buffer and PIC. After lysate was sonicated and centrifuged, 50 μl supernatant was taken for chromatin digestion. 10 μl digested chromatin was used as the “Input”, and the rest was subjected to the following immunoprecipitation experiments. Normal rabbit IgG (Invitrogen) was used as the negative control. ChIP PCRs were performed using primers that flank the G-box-like motif sites and primers that do not flank the sites in the promoter regions of target genes as controls. Primers (Comate Bioscience Technology) for ChIP are listed in Supplementary Table S2. The quantitative analysis of the ChIP gel electrophoresis map was carried out by Image J (National Institutes of Health).

### Luciferase assays

ELIP1 cDNA (603 bp) was amplified by PCR and cloned into pCambia1301vector. The 5′ promoter fragments of *GUN4* (1672 bp), *HEMA1* (450 bp), and *CHLM* (1313 bp) were separately cloned into the pGreenII 0800-Luc vectors. The mutagenized vectors were constructed by PCR-based site-directed mutagenesis. Restriction enzyme-treated PCR products were transformed into *Escherichia coli*. Positive clones were selected and verified by gene sequencing.

One microgram each of *CHLM-Luc, GUN4-Luc*, and *HEMA1-Luc* reporter vectors and 1 μg ELIP1 plasmid were transformed by agrobacterium-sensitive cells and injected into the 1-month-old tobacco blade. PRL-TK vector was added for control. Then, the same amount of injected tobacco blade was adopted, fully ground, and added with the cell lysate to divide the tobacco blade. Then, cells of the cracked tobacco blade were collected. One hundred milliliters each of the sample and luciferase reagent was added, and luciferase reporter assays were done using a dual-reporter assay system (Promega, E1980).

### Data analyses

Results correspond to mean ± SD of the quantification of three independent experiments. Asterisks indicate a significant difference compared to control negative as analyzed by Dunnett, one-way ANOVA. (*p < 0.05; **p < 0.01; ***p < 0.001).

### Statement

The study comply with local and national guidelines.

## Supplementary Information

Supplementary Information 1.

Supplementary Information 2.

## Data Availability

All data generated or analyzed during this study are included in this published article and its supplementary information files.
