# Supplementary material for: Light regulates chlorophyll biosynthesis via ELIP1 during the storage of Chinese cabbage
Source: Sci Rep. 2022 Jun 30;12:11098. doi: 10.1038/s41598-022-15451-9 (PMC9247097; doi:10.1038/s41598-022-15451-9)
Supplement: Supplementary file 1 — Supplementary Information 1. [file 41598_2022_15451_MOESM1_ESM.doc]

**Light regulates chlorophyll biosynthesis via ELIP1 during the storage of Chinese cabbage**

Tuoyi Wang^1^*, Sijia Liu^1^, Shaonan Tian^1^, Tianyi Ma^2^, Wei Wang^1^

*^1^ College of Food and Biological Engineering, Qiqihar University, Qiqihar 161006, China*

*^2^ College of Life Sciences, Agriculture and Forestry, Qiqihar University, Qiqihar 161006, China*

* Corresponding author: Tuoyi Wang.

Email: wangtuoyi@hotmail.com

**Table S1. List of primers** **for RT-qPCR**

| Gene name | Acc.No. | Notes | Description | Sequence（5'→3'） |
| --- | --- | --- | --- | --- |
| ELIP1 | NM_113183.4 | RT-qPCR | Forward | TATGGCTGAGGGAGAACCCA |
|  |  | RT-qPCR | Reverse | CGCTGAACGCTAACAAGTCG |
| GUN4 | NM_115802.4 | RT-qPCR | Forward | TCTATTTGGACACGGGCCAC |
|  |  | RT-qPCR | Reverse | AGAGTGGTTCTACGCTTCACT |
| HEMA1 | NM_104609.4 | RT-qPCR | Forward | TTTGGGTCCGGTTTGGTTCA |
|  |  | RT-qPCR | Reverse | GCGTGGAAGTAGAAAGGCCA |
| LHCB1 | NM_102732.3 | RT-qPCR | Forward | CTGCGTCTTCCCTGAGTTGT |
|  |  | RT-qPCR | Reverse | ATGCTCTGAGCGTGAACCAA |
| LHCB2 | NM_126540.4 | RT-qPCR | Forward | AAGAACGGACGTCTTGCCAT |
|  |  | RT-qPCR | Reverse | GTAGCGTAAGACCAGGCGTT |
| ACTIN2* | NM_112764.4 | RT-qPCR | Forward | CTTGCACCAAGCAGCATGAA |
|  |  | RT-qPCR | Reverse | CCGATCCAGACACTGTACTTCCTT |
| CHLM | NM_001341721.1 | RT-qPCR | Forward | GGCTTATCTACACGCGGAGG |
|  |  | RT-qPCR | Reverse | GGAACAGCTTCGATGAGCCT |

*(c) is control primer.

**Table S2. List of primers for ChIP assay**

| Gene name | Acc.No. | Notes | Description | Sequence（5'→3'） |
| --- | --- | --- | --- | --- |
| CHLM1 | NM_001341721.1 | ChIP | Forward | CCATTACAATACACCTAACGGTTT |
|  |  | ChIP | Reverse | GCATCAGCTTCCTCCACACT |
| CHLM2 | NM_001341721.1 | ChIP | Forward | TCACTGCCGAGTCCAAGTTC |
|  |  | ChIP | Reverse | AAACCGTTAGGTGTATTGTAATGG |
| CHLM3 | NM_001341721.1 | ChIP | Forward | AAAACCCTTGAGTTTCCAGCC |
|  |  | ChIP | Reverse | CCGTCTCTGGTTTTGCCTGT |
| HEMA1 | NM_104609.4 | ChIP | Forward | TTTGGGTCCGGTTTGGTTCA |
|  |  | ChIP | Reverse | GCGTGGAAGTAGAAAGGCCA |
| GUN4 | NM_115802.4 | ChIP | Forward | TCTATTTGGACACGGGCCAC |
|  |  | ChIP | Reverse | AGAGTGGTTCTACGCTTCACT |

**Table S3. List of Chlorophyll a/b-binding genes screening from DEGs**

| **No.** | **Gene ID** | **Gene name and functions** | ***p-value***  **T01_VS_T02** | ***p-value* T01_VS_T03** | ***p-value* T02_VS_T02** |
| --- | --- | --- | --- | --- | --- |
| 1 | Bra033911 | ELIP1, ELIP; ELIP1 (EARLY LIGHT-INDUCABLE PROTEIN); chlorophyll binding | -2.62888 | -5.78879 | -3.22942 |
| 2 | Bra013183 | LHCB2.1, LHCB2; LHCB2.1; chlorophyll binding | 4.428445 | 2.990623 | -1.4812 |
| 3 | Bra010807 | CAB2, AB165, LHCB1.1; CAB2 (CHLOROPHYLL A/B-BINDING PROTEIN 2); chlorophyll binding | 5.035103 | 2.542906 | -2.53749 |

DEGs is the differentially expressed genes.


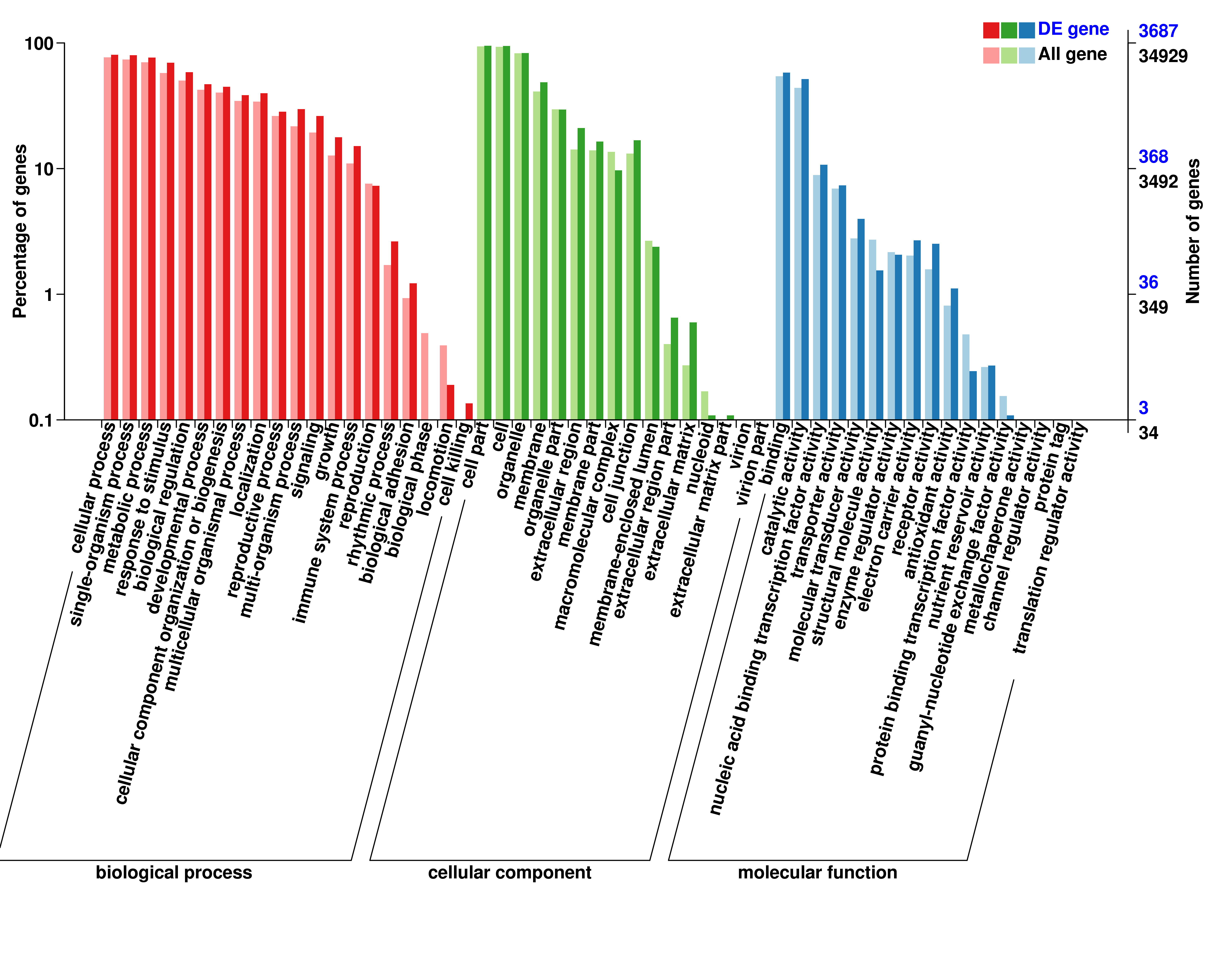


**Figure S1 - Differentially expressed genes analyses by GO and KEGG at T01.** TS01 represents the fresh samples on the first day. GO is Gene Ontology; KEGG is Kyoto Encyclopedia of Genes and Genomes.


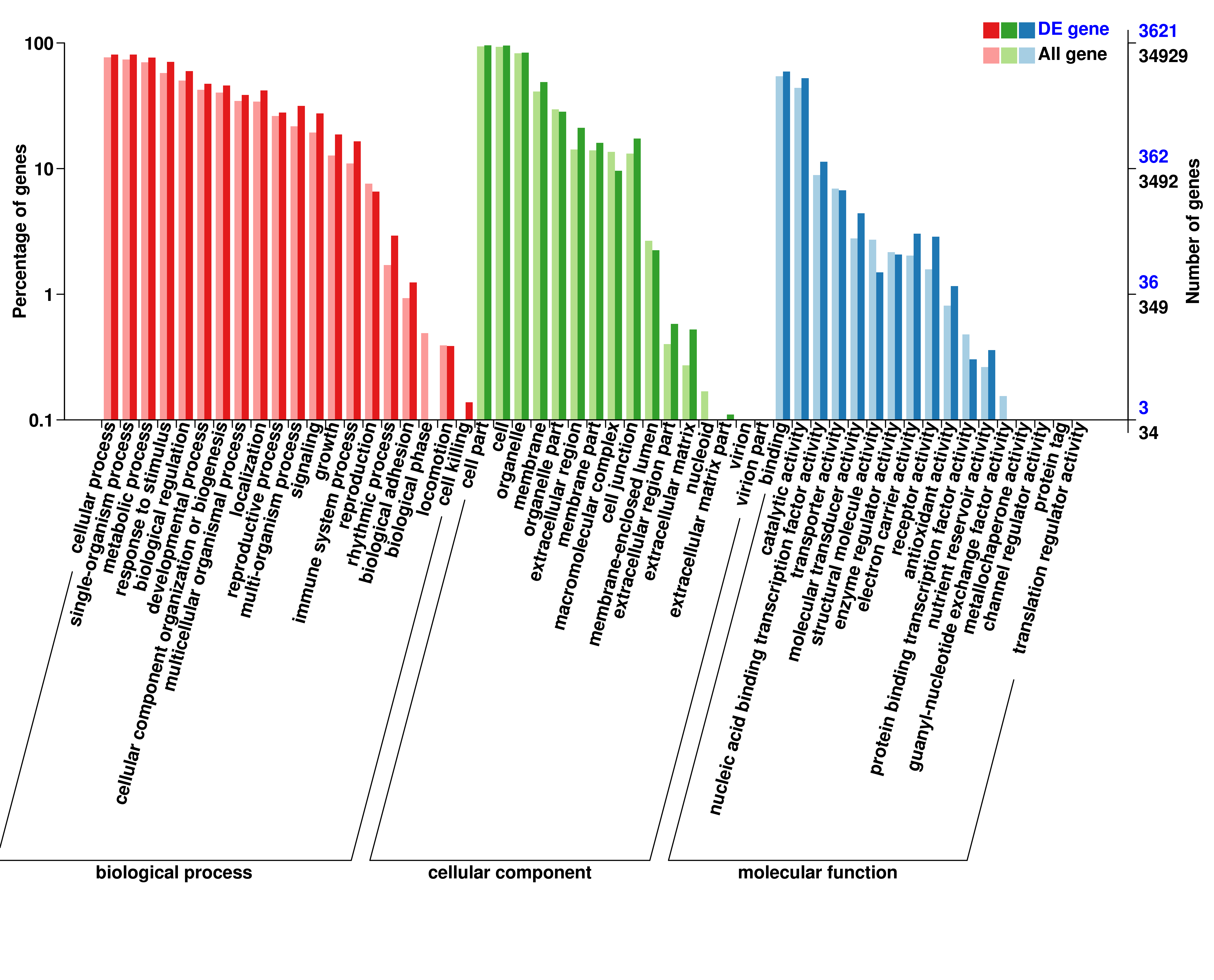


**Figure S2 - Differentially expressed genes analyses by GO and KEGG at T02.** TS02 represents the samples after ten days’ dark storage.


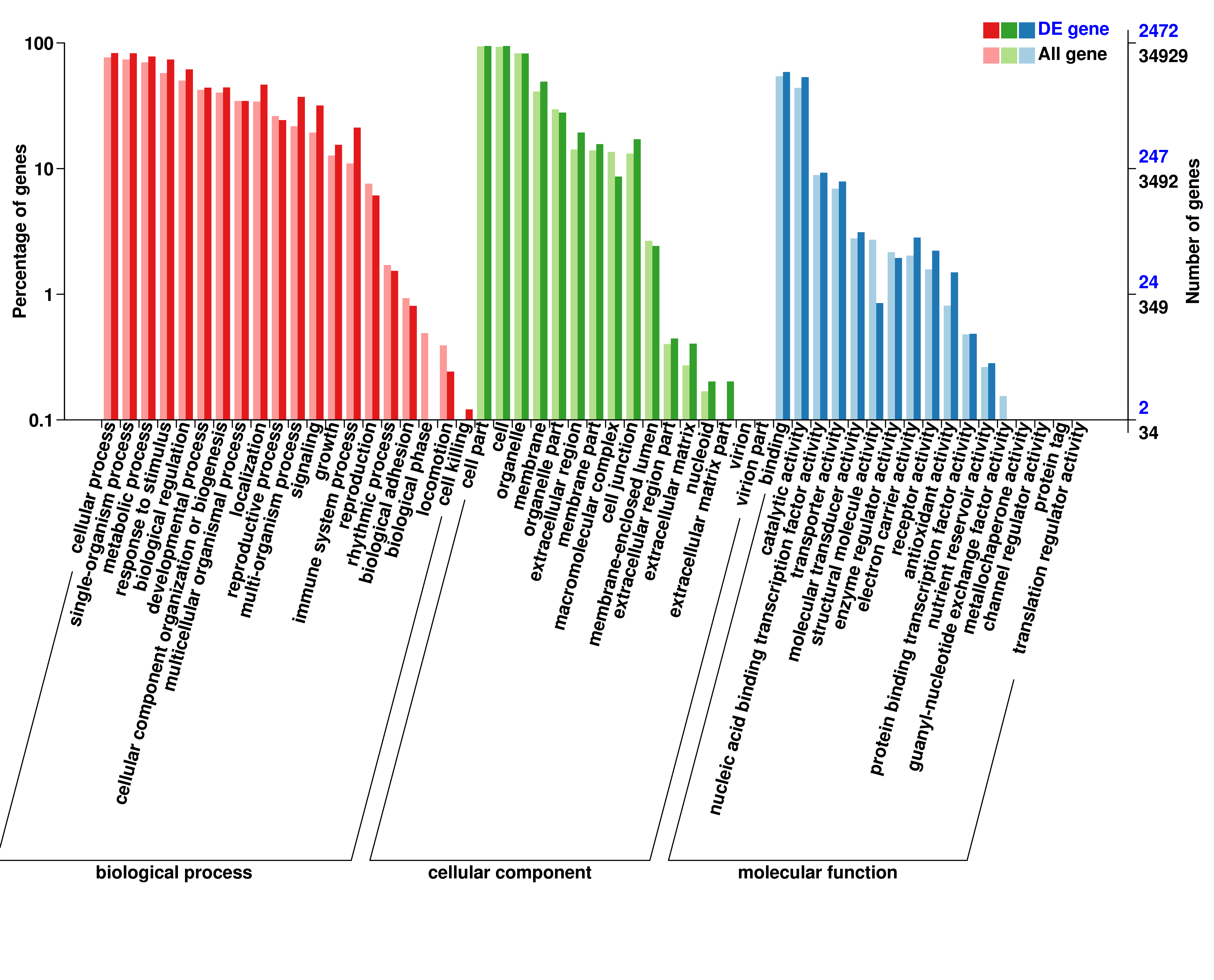


**Figure S3 - Differentially expressed genes analyses by GO and KEGG at T03.** TS03 represents the samples after twenty days’ dark storage.


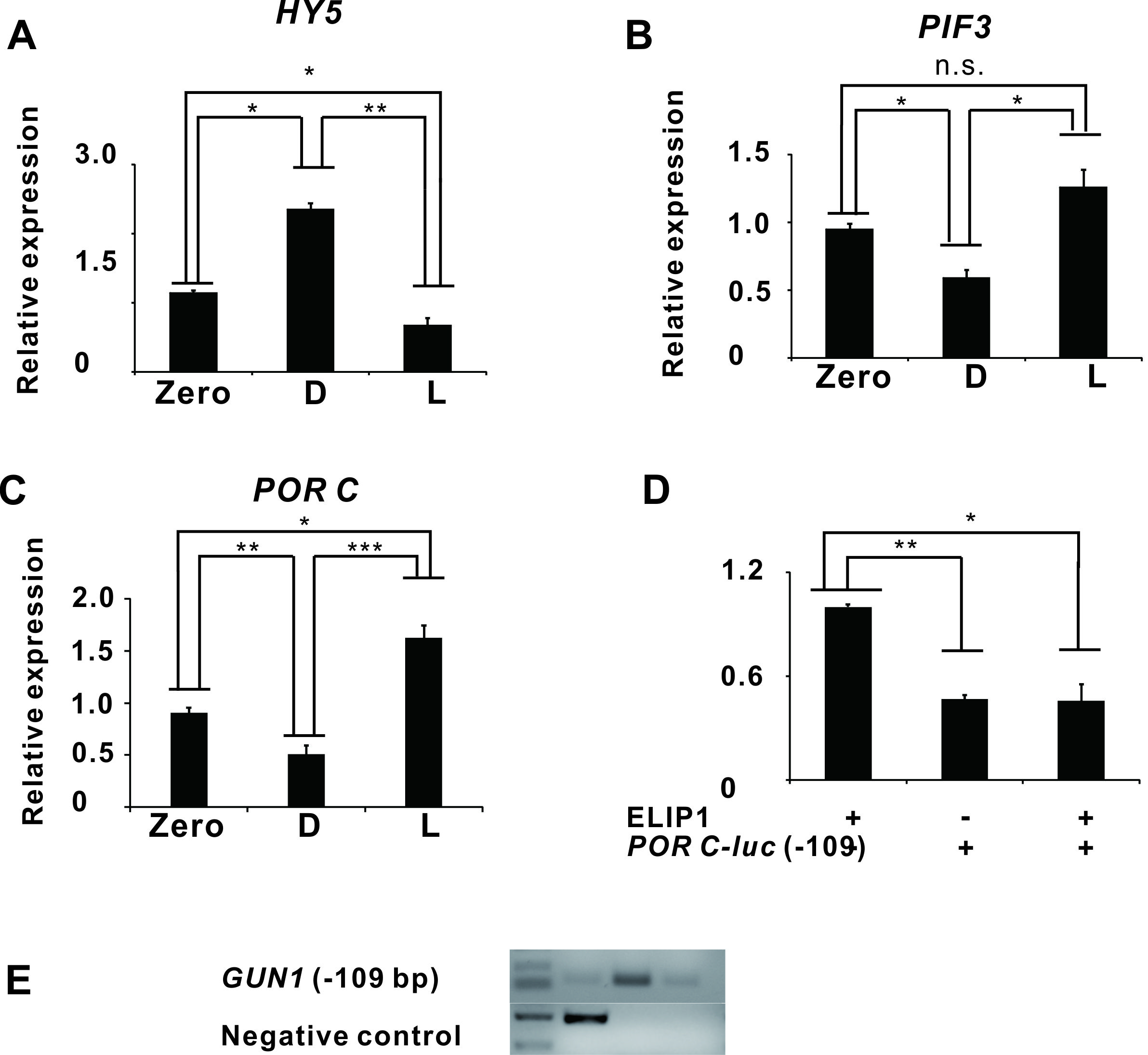


**Figure S4 - Relative genes expression analyses. (A)** *HY5* expression. **(B)** *PIF3* expression. **(C)** *POR C* expression. **(D)** Luciferase reporter assays. **(E)** ChIP assays. Asterisks indicate a significant difference compared to control negative as analyzed by Dunnett, one-way ANOVA. (* P<0.05; ** P<0.01; *** P<0.001). All error bars are expressed as SEM.
