# Supplementary material for: Light regulates chlorophyll biosynthesis via ELIP1 during the storage of Chinese cabbage
Source: Sci Rep. 2022 Jun 30;12:11098. doi: 10.1038/s41598-022-15451-9 (PMC9247097; doi:10.1038/s41598-022-15451-9)
Supplement: Supplementary file 2 — Supplementary Information 2. [file 41598_2022_15451_MOESM2_ESM.doc]

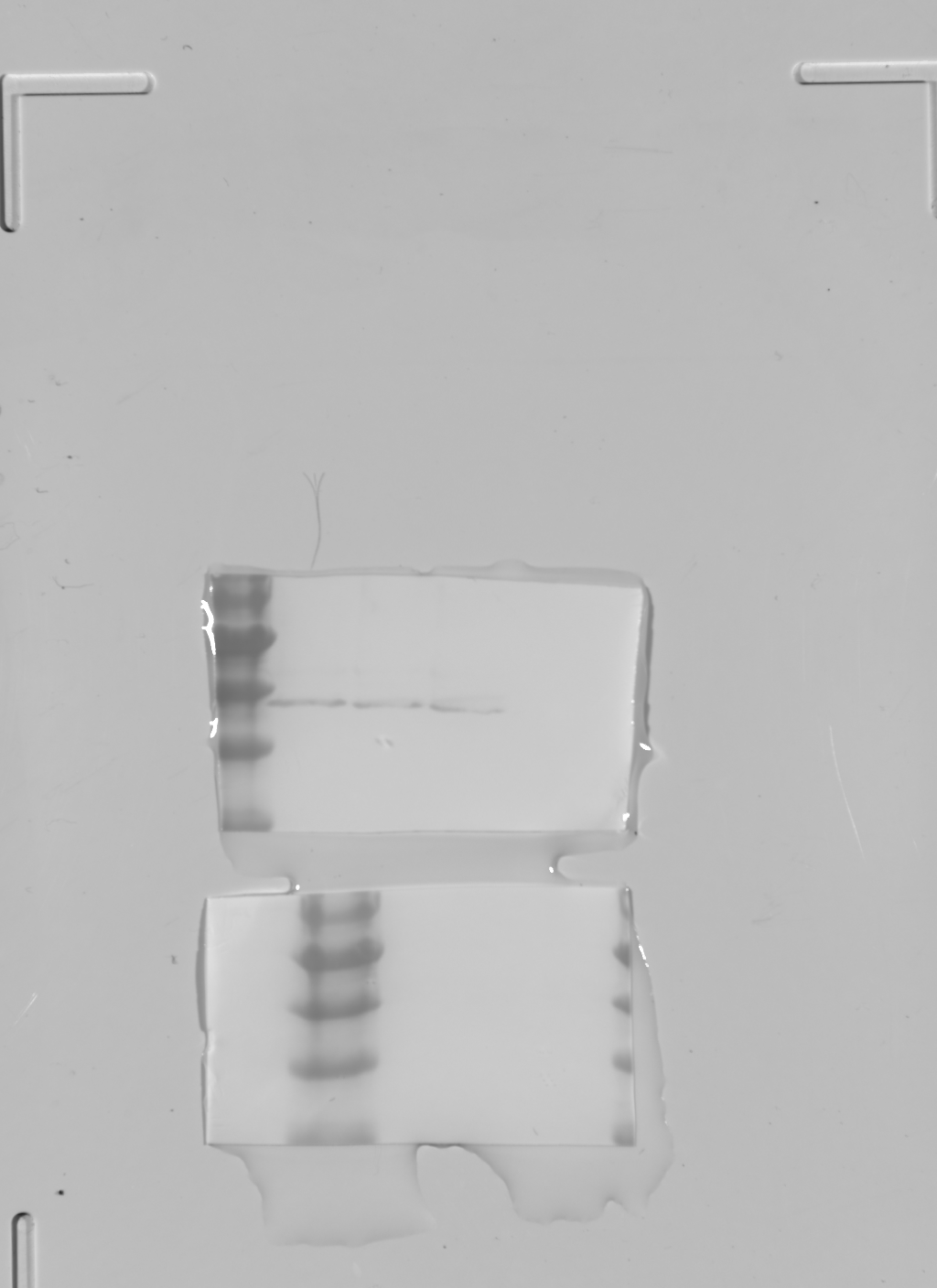

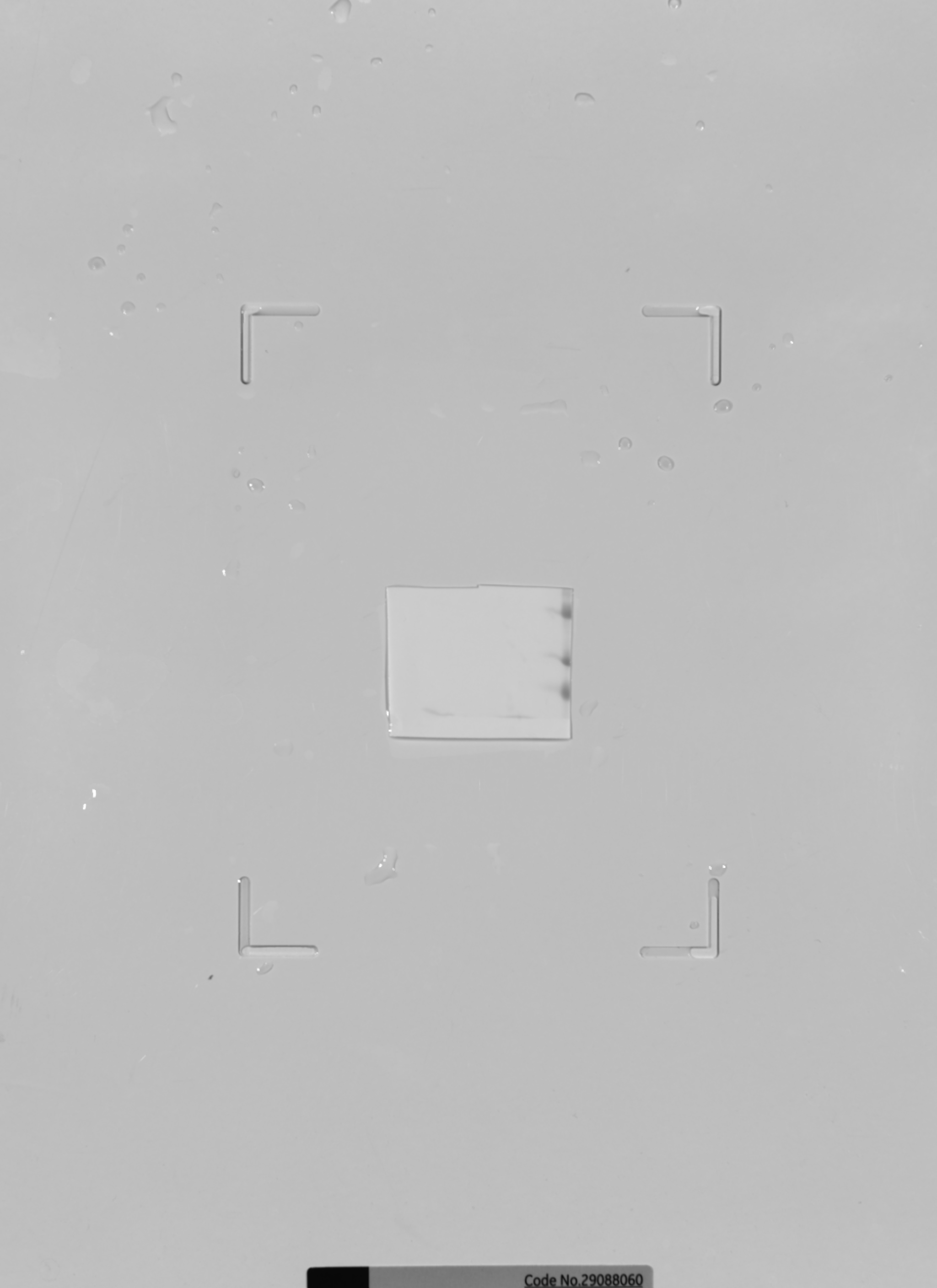


Actin for WB (Fig 2B)

ELIP1 for WB (Fig 2B)


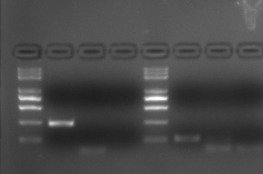


Negative control for ChIP (Fig 4B)


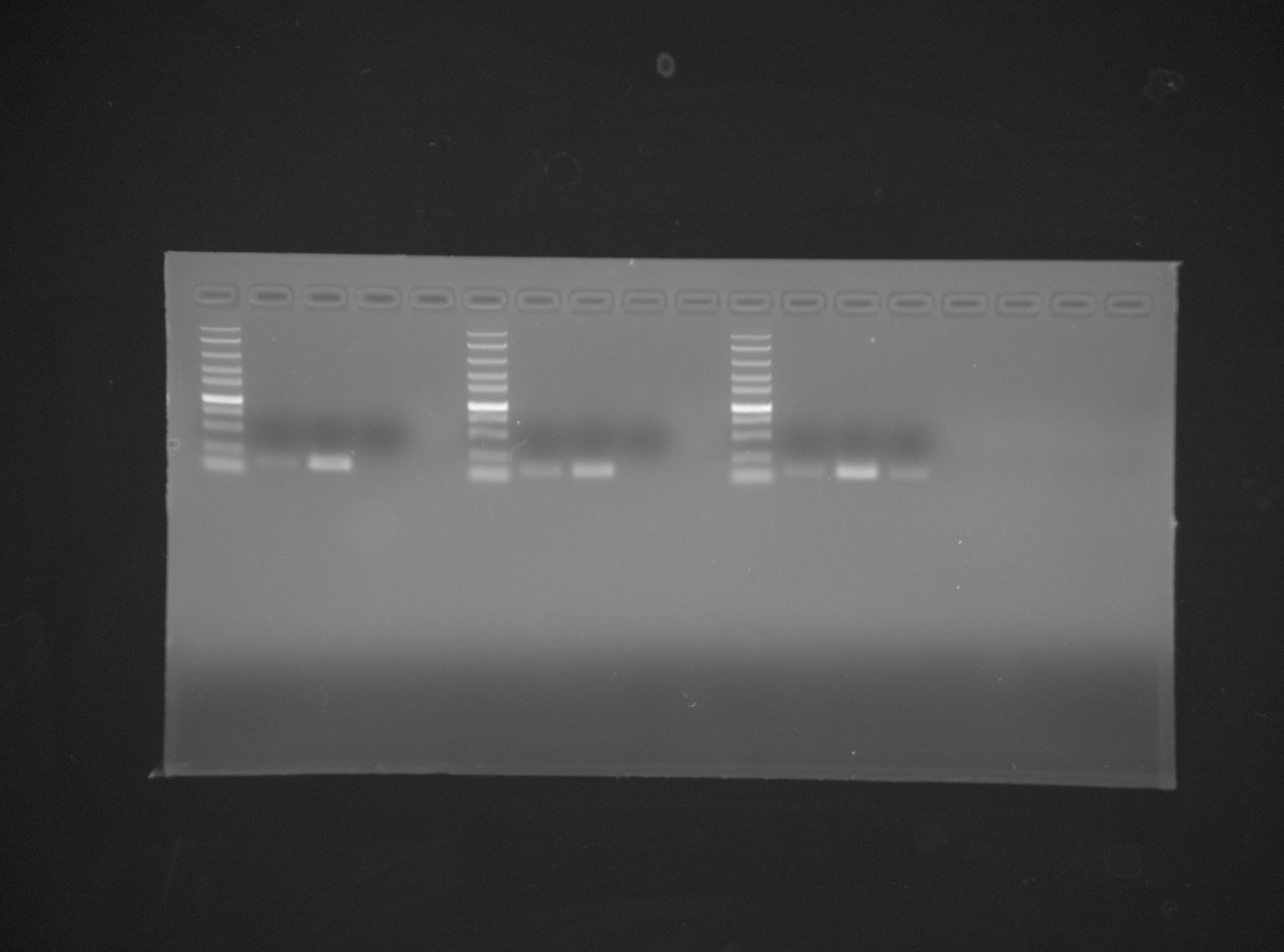

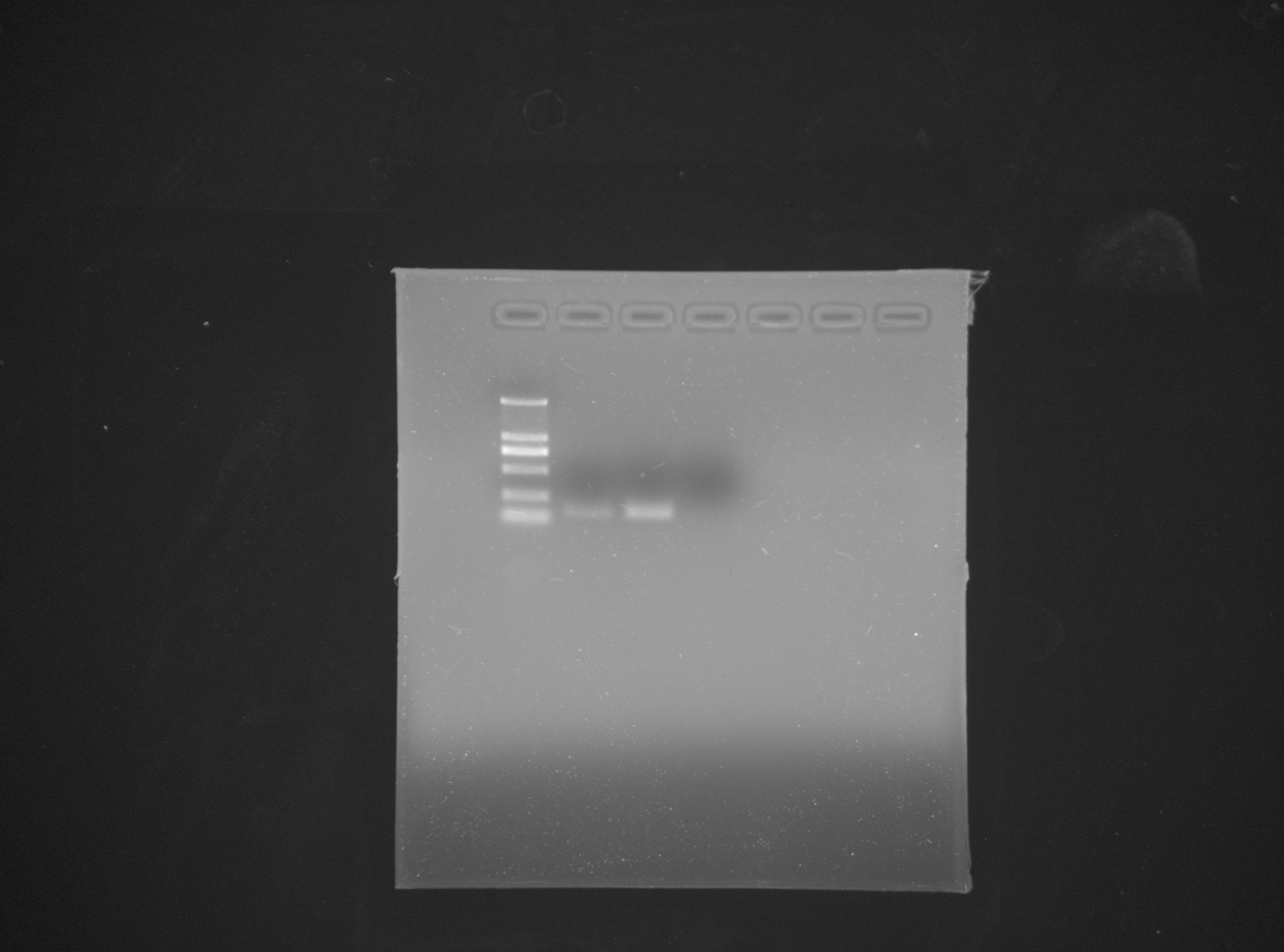

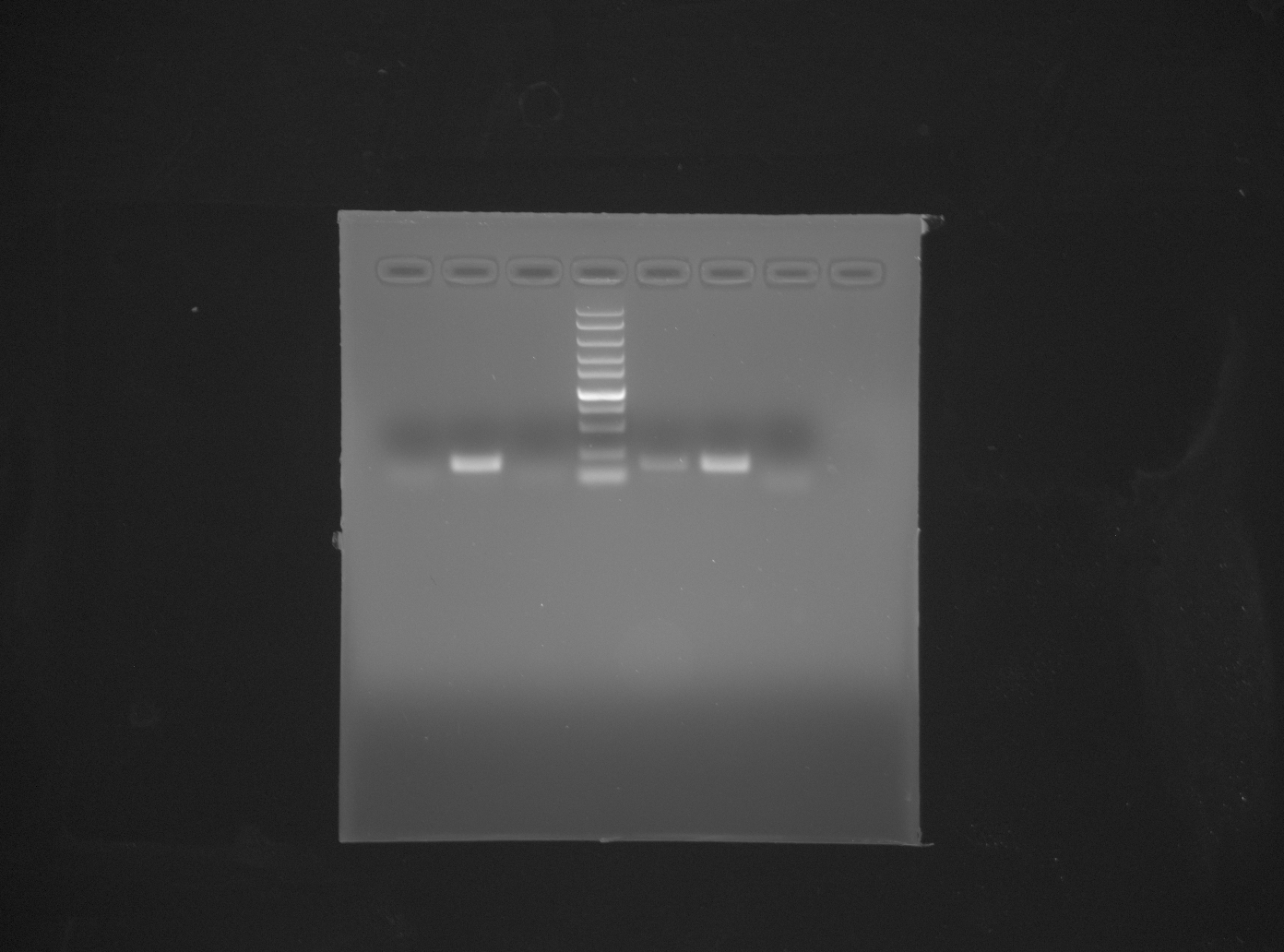


*CHLM1* (-1,034 bp) (Fig 4B)

*CHLM1* (-1,034 bp) (Fig 4B)

*POR C* (-109 bp) (Fig S4E)

*GUN4* (-1,568 bp) (Fig 4B)

*HEAM1* (-168 bp) (Fig 4B)
